# Transcriptome Analysis and RNA Interference Reveal *GhGDH2* Regulating Cotton Resistance to Verticillium Wilt by JA and SA Signaling Pathways

**DOI:** 10.3389/fpls.2021.654676

**Published:** 2021-06-11

**Authors:** Xian-Peng Xiong, Shi-Chao Sun, Qian-Hao Zhu, Xin-Yu Zhang, Feng Liu, Yan-Jun Li, Fei Xue, Jie Sun

**Affiliations:** ^1^Key Laboratory of Oasis Eco-Agriculture, College of Agriculture, Shihezi University, Shihezi, China; ^2^Shenzhen Branch, Guangdong Laboratory for Lingnan Modern Agriculture, Genome Analysis Laboratory of the Ministry of Agriculture, Agricultural Genomics Institute at Shenzhen, Chinese Academy of Agricultural Sciences, Shenzhen, China; ^3^CSIRO Agriculture and Food, Canberra, ACT, Australia

**Keywords:** *Gossypium hirsutum*, Verticillium wilt, transcriptome, weighted gene co-expression network analysis, *GhGDH2*, jasmonic acid, salicylic acid

## Abstract

Verticillium wilt, caused by *Verticillium dahliae*, is one of the most damaging and widespread soil-borne cotton diseases. The molecular mechanisms underlying the cotton defense against *V. dahliae* remain largely elusive. Here, we compared the transcriptional differences between Upland cotton cultivars: one highly resistant (HR; Shidalukang 1) and one highly susceptible (HS; Junmian 1). This was done at multiple time points after *V. dahliae* inoculation, which identified 2010 and 1275 differentially expressed genes (DEGs) in HR and HS, respectively. Plant hormone signal transduction-related genes were enriched in HR, whereas genes related to lignin biosynthesis were enriched in both HR and HS. Weighted gene co-expression network analysis (WGCNA) using the 2868 non-redundant genes differentially expressed between the *V. dahliae* infected and uninfected samples in HR or HS identified 10 different gene network modules and 22 hub genes with a potential role in regulating cotton defense against *V. dahliae* infection. *GhGDH2*, encoding glutamate dehydrogenase (GDH), was selected for functional characterization. Suppressing the expression level of *GhGDH2* by virus-induced gene silencing (VIGS) in HS led to inhibition of the salicylic acid (SA) biosynthesis/signaling pathways and activation of the jasmonic acid (JA) biosynthesis/signaling pathways, which resulted in an increase of 42.1% JA content and a reduction of 78.9% SA content in cotton roots, and consequently enhanced *V. dahliae* resistance. Our finding provides new insights on the molecular mechanisms of cotton resistance to *V. dahliae* infection and candidate genes for breeding *V. dahliae* resistance cotton cultivars by genetic modification.

## Introduction

Cotton (*Gossypium hirsutum* L.) is an economically important crop of the *Malvaceae* family, providing a source of renewable natural fiber and oilseeds. Cotton is continuously subjected to various diseases during its growth and development, which influence the yield and quality of cotton fiber. *Verticillium dahliae* is a soil-borne fungus and cause of vascular disease known as Verticillium wilt, which is one of the most common and serious diseases in cotton-producing regions worldwide (Cai et al., 2009). *V. dahliae* can infect cotton by penetrating roots during the whole growing season and result in wilting and defoliation of leaves, and even plant death (Shaban et al., 2018). Breeding resistant cultivars are the most effective, economical, safe, and environmentally friendly method for controlling Verticillium wilt. Due to the lack of disease-resistant germplasm resources in *G. hirsutum* and variation in *V. dahliae* strains, the breeding progress on Verticillium wilt resistance is slow (Wang et al., 2016). Understanding the molecular mechanism of Verticillium wilt resistance and identifying Verticillium wilt resistance genes are of great significance for improving Verticillium wilt resistance in cotton by genetic engineering.

*Gossypium barbadense* is more resistant to *V. dahliae* than *G. hirsutum* (Zhang et al., 2013). Previous studies have shown that a few major genes control the resistance of *G. barbadense* to *V. dahliae*, whereas *V. dahliae* resistance in *G. hirsutum* seems to be controlled by multiple genes (Zhang et al., 2013; Li et al., 2017). Transcriptomic analysis is an effective and widely used approach to identify genes differentially expressed in different biological processes or response to environmental stresses (Wang et al., 2009). One of the powerful approaches to investigate the mechanisms of cotton resistance to *V. dahliae* is to analyze the basal transcriptome and its response to *V. dahliae* infection in cotton cultivars with different levels of resistance. RNA-sequencing studies with an interest on the mechanisms of cotton – *V. dahliae* interaction have been carried out using various *V. dahliae* strains in different cotton species (Xu et al., 2011b; Sun et al., 2013; Zhang et al., 2013, 2016; Dong et al., 2019; Li C. et al., 2019), but only one study compared the transcriptomic differences between resistant and susceptible *G. hirsutum* varieties (Zhang et al., 2017), in which phenylpropanoid biosynthesis and various plant hormone signaling pathways were found to play an important role in *G. hirsutum* defense against *V. dahliae*.

Previous studies on the interaction between plants and *V. dahliae* found changes in phenylpropanoid biosynthesis in plants after *V. dahliae* infection, in particular, the lignin biosynthesis pathway was found to be activated (Xu et al., 2011b; Zhang et al., 2013; Dhar et al., 2020). Inhibition of the expression of the lignin biosynthesis related genes *GhHCT1* and *GhLac1* enhanced susceptibility of cotton to *V. dahliae* due to decreased activity of lignin biosynthesis (Guo et al., 2016; Hu et al., 2018). Several genes such as *GhERF1*, *GhARPL18A-6*, *GhCOMT*, and *GhSNAT1* have been shown to positively mediate cotton resistance to *V. dahliae* by increasing lignin content (Guo et al., 2016; Li et al., 2019; Zhang et al., 2019). Jasmonic acid (JA) and salicylic acid (SA) are the two important defense signal molecules in plant immune response (Thaler et al., 2012). SA and JA play a key role in resistance against biotrophic and necrotrophic pathogens, respectively. Antagonistic interaction between SA and JA in defense response has been well documented in *Arabidopsis* (Thaler et al., 2012). Various studies have revealed that both JA and SA positively regulate cotton defense against *V. dahliae* infection (Shaban et al., 2018; Dhar et al., 2020). However, it is still unclear whether SA and JA have an antagonistic relationship in the process of cotton response to *V. dahliae* infection.

To explore the molecular mechanisms associated with *V. dahliae* resistance in *G. hirsutum*, we have sequenced the transcriptomes of Shidalukang 1 (HR; *V. dahliae*-resistant) and Junmian 1 (HS; *V. dahliae*-susceptible) at different time points after *V. dahliae* infection. In our previous investigation, we found that the basal transcriptome landscape between HR and HS has a substantial role in their responses to *V. dahliae* infection and that *V. dahliae* resistance is affected by the metabolites of the lignin biosynthesis pathway (Xiong et al., 2021). Here, we focused on the changes of transcriptomes in response to *V. dahliae* infection in HR and HS, and identified significantly enriched gene ontology (GO) terms and Kyoto Encyclopedia of Genes and Genomes (KEGG) pathways using the genes induced or repressed by *V. dahliae* infection in HR and HS. Moreover, 22 hub genes associated with cotton response to *V. dahliae* infection were identified by weighted gene co-expression network analysis (WGCNA), and the function of one (*GhGDH2*) of the hub genes was characterized through virus-induced gene silencing (VIGS). This work enriched our knowledge on the molecular-defense networks of *G. hirsutum* in response to *V. dahlia*e infection and identified candidate genes for enhancing cotton resistance to *V. dahliae*.

## Materials and Methods

### Plant Materials and *Verticillium dahliae* Inoculation

Two *G. hirsutum* varieties, Shidalukang 1 (*V. dahliae*-resistant; HR) and Junmian 1 (*V. dahliae*-susceptible; HS), from our own seed stock were used in RNA-seq analysis. Root and stem samples were collected at 0 (control), 12, 24, and 48 h after inoculated with *V. dahliae* and a mixture of 200 mg roots and 200 mg stems was used in RNA extraction and RNA-seq. The VIGS experiments and disease assays were carried out using HS. Plants were cultivated in a greenhouse under 24/22°C and a photoperiod scheme of 16-h light/8-h dark. Disease assays were performed using the highly aggressive defoliating *V. dahliae* strain V991 kindly provided by Associate Professor Yanfei Sun of the Shihezi University. Two-leaf stage seedlings were inoculated with *V. dahliae* by the root irrigation method (Xiong et al., 2019).

### Transcriptome Analysis

Construction and sequencing of RNA-seq libraries (24 in total, i.e., 4 time points × 2 cultivars × 3 biological replicates) from HR and HS have been previously described (Xiong et al., 2021). The raw short-reads containing adapter and/or ploy-N and low quality were removed using the Trimmomatic v0.30 program (Bolger et al., 2014). All the clean reads were aligned to the TM-1 genome (Zhang et al., 2015) using TopHat v2.0.12. Genes induced or repressed after *V. dahliae* inoculation in HR and HS were identified using the R package DESeq2 (Love et al., 2014). FPKM (fragments per kilobase of transcript sequence per million base pairs sequenced) was used to represent gene expression level (Trapnell et al., 2010). Genes with a | log2 (fold-change)| ≥ 1 and an adjusted *P* value < 0.01 were considered to be differentially expressed. Venn diagram and heatmap were generated using the Novemagic server^1^. The raw data of RNA-seq are available in the National Center for Biotechnology Information (NCBI) under the accession no. PRJNA593765.

A total of 2010 and 1,275 differentially expressed genes (DEGs) from HR and HS were used in analyses of GO terms and KEGG pathways by the GOseq and KOBAS 2.0 R package, respectively (Young et al., 2010; Xie et al., 2011). GO terms and KEGG pathways with a corrected *P* value < 0.05 were regarded as significant and selected for further analysis.

### Co-expression Network Analysis

The FPKM of the 2868 non-redundant DEGs from HR and HS was used to construct the co-expression networks by the R package WGCNA (Langfelder and Horvath, 2008). First, the network was constructed in accordance with the scale-free topology criterion using a β of 14 as a weight function. The co-expression modules were identified with the default settings, except that the TOMType was assigned, and minModuleSize and merge CutHeight were set to 30 and 0.25, respectively. Each module was assigned by a unique color; genes not assigned to any module were grouped into the gray module.

### Salicylic Acid and Methyl Jasmonate Treatments

For the SA and methyl jasmonate (MeJA) treatment experiments, two-leaf stage seedlings of Shidalukang 1 were sprayed by 1 mmol L^–1^ SA or 100 μmol L^–1^ MeJA. Root samples were collected from five individual cotton seedlings at 0, 12, 24, 48, and 72 h after treatments. Moreover, the roots, stems, and leaves from five individual seedlings of Shidalukang 1 or Junmian 1 at two-leaf stage were collected to measure the expression profiles of *GhGDH2* and *GhGDH3*. Three independent biological replicates were sampled at each time points for both SA and MeJA treatments.

### RNA Extraction and Real-Time Quantitative PCR Analysis

Total RNA was isolated using an RN09-EASYspin RNA Plant Mini Kit (Aidlab, Beijing, China) from roots, stems, and leaves collected from seedlings treated with SA, MeJA, or *V. dahliae*, respectively. The quality and concentration of the extracted RNA were determined by a NanoPhotometer spectrophotometer (IMPLEN, CA, United States). Total RNA (3 μg) was used in synthesis of cDNA template using the PrimeScript II 1st Strand cDNA Synthesis Kit (TaKaRa, Dalian, China). Then, 2 μl of a 10-times diluted cDNA was used in quantitative PCR (qPCR) using the SYBR Green kit (Roche, Rotkreuz, Switzerland) on a Light-Cycler 480II (Roche). The qPCR program comprised an initial denaturation step at 95°C for 10 min, followed by 40 cycles at 95°C for 15 s and 60°C for 15 s, and 72°C for 15 s. qPrimerDB^2^ was used to design the real-time quantitative PCR (RT-qPCR) primers (Supplementary Table 1). *GhUBQ7* (DQ116441.1) was used as the internal control and the relative gene expression level from three biologically independent samples was evaluated using the 2^–Δ^ ^Δ^ ^*CT*^ method (Livak and Schmittgen, 2001).

### Virus-Induced Gene Silencing

Tobacco rattle virus (TRV)-based vectors were used in the VIGS experiments to explore the function of *GhGDH2* in cotton defense against *V. dahliae*. *TRV*:*GhCHLI* was used as a positive control for the VIGS experiment as previously reported (Xiong et al., 2019). A 405-bp fragment specific to *GhGDH2* (*Gh_D03G0992*) was amplified by PCR from HR with gene-specific primers (Supplementary Table 1). VIGS was performed with the same procedures as previously described (Gao et al., 2013a). Approximately 14 days after infiltration, the roots of five *TRV:00* and *TRV:GhGDH2* plants were collected to measure the expression level of *GhGDH2* by RT-qPCR. The RT-qPCR primer sequences for *GhGDH2* are listed in Supplementary Table 1. Thirty *TRV:00* and *TRV:GhGDH2* plants at the two-leaf-stage were subjected to *V. dahliae* inoculation to compare their disease response.

### Evaluation *Verticillium dahliae* Resistance of VIGS Cotton Plants

The ratio of diseased plants and disease index were calculated using thirty *TRV:00* and *TRV2:GhGDH2* plants at 14 and 21 days post-infection (dpi) according to the following formula: ratio of diseased plants = (the number of diseased plants/the total number of inoculated plants) × 100%. Disease index = [(Σ disease grade × the number of plants with the corresponding disease grade)/(4 × the total number of inoculated plants)] × 100. Cotton seedlings were classified into five grades (i.e., grades 0, 1, 2, 3, and 4) according to the phenotypes on the true leaves of *TRV:00* and *TRV2:GhGDH2* plants after *V. dahliae* infection (Gao et al., 2013a). The final results were the average of three independent experiments.

The stems above cotyledons of *TRV:00* and *TRV2:GhGDH2* plants were hand-sectioned at 14 dpi and observed using a stereomicroscope (SteREO Discovery.V20; ZEISS). For fungal recovery assay, ten 1-cm stem sections above the cotyledons of cotton plants were cut at 14 dpi and surface sterilized in 3% hypochlorite for 3 min. After rinsing three times with sterile water, the stem sections were plated on PDA medium for a week at 25°C in darkness. The relative fungal biomass was determined as previously described (Ellendorff et al., 2009). Briefly, the stems above the cotyledons of *TRV:00* and *TRV2:GhGDH2* plants were collected at 14 and 21 dpi for DNA extraction. *V. dahliae* specific primers ITS1-F and ST-Ve1-R and primers for the cotton *UBQ7* gene were used to quantify fungal biomass by qPCR (Supplementary Table 1).

### Measurement of JA and SA Content

The contents of endogenous SA and JA were measured as described previously with minor modifications (Sun et al., 2014). Briefly, at 72 h post-inoculation (hpi), root samples were collected from mock (inoculated with sterile water) and *V. dahliae*-inoculated *TRV:00* and *TRV:GhGDH2* plants. Approximately 200 mg of fresh root sample was ground to powder with liquid nitrogen and then extracted twice with 2 ml pre-cold 80% methanol overnight under dark at 4°C. The supernatant was dried by N_2_ at room temperature, then re-dissolved in 0.4 ml methanol. After filtering through a 0.22-μm membrane, the collected supernatant was used in quantification of the content of SA and JA by the high-performance liquid chromatography–tandem mass spectrometry (HPLC/MS) system (AB SCIEX Triple Quad 5500). Pure SA or JA (both ordered from Sigma) was used as a respective internal standard.

### Data Analysis

SPSS (version 23.0; IBM, United States) was used for the statistical analysis. Data were checked for normality and homogeneity of variances with the Shapiro–Wilk test (α = 0.05) and the Levene test (α = 0.05), respectively. The differences between groups were compared using Student’s *t*-test (^∗^*P* < 0.05; ^∗∗^*P* < 0.01).

## Results

### Differentially Expressed Genes Upon *Verticillium dahliae* Infection

**FIGURE 1:**
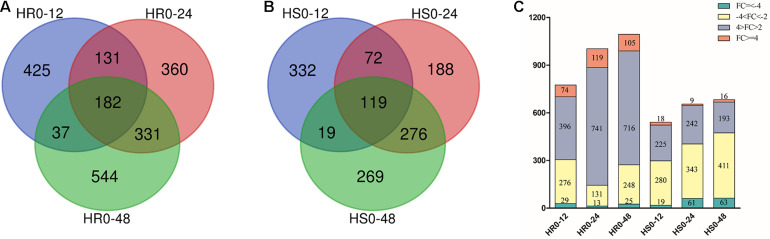
Comparison of the number of genes differentially expressed in Shidalukang 1 (HR) and Junmian 1 (HS) at 12, 24, and 48 h after *Verticillium dahliae* infection. **(A,B)** The number of differentially expressed genes (DEGs) unique to or overlapping between each time point in **(A)** HR and **(B)** HS following *V. dahliae* infection. **(C)** The number of DEGs with different fold-change in HR and HS after *V. dahliae* infection.

To characterize the genes responsive to *V. dahliae* infection in cotton cultivars with different levels of resistance, we compared the expression level of genes in the 12, 24, and 48 hpi samples with that in the uninfected sample (0 hpi). Based on the criteria of adjusted *P* value < 0.01 and | log2 (fold change)| ≥ 1, 775, 1004, and 1094 DEGs were observed at 12, 24, and 48 hpi in HR, respectively (Figure 1A), and 542, 655, and 683 DEGs were found at 12, 24, and 48 hpi in HS, respectively (Figure 1B). In total, 2010 and 1,275 non-redundant DEGs were identified in HR and HS, respectively (Supplementary Tables 2, 3). Among them, 182 and 119 genes were induced or repressed by *V. dahliae* infection at all three time points in HR and HS, respectively (Figures 1A,B). The number of DEGs was 48 hpi > 24 hpi > 12 hpi in both HR and HS, and with more DEGs in HR than in HS at each time point (Figure 1C). HR had more induced genes than repressed genes at all three time points, but HS showed the opposite trends (Figure 1C). Overall, 2868 non-redundant DEGs were detected in HR and HS with 417 DEGs common in both cultivars (Supplementary Figure 1 and Supplementary Table 4).

### Functional Annotation of DEGs

**FIGURE 2:**
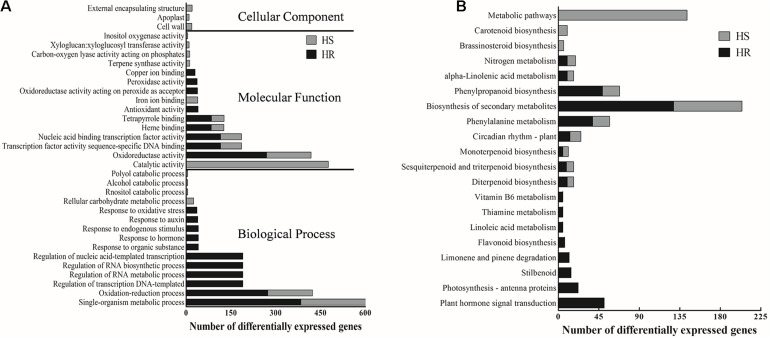
Gene ontology (GO) and Kyoto Encyclopedia of Genes and Genomes (KEGG) analysis of the 2010 and 1,275 differentially expressed genes (DEGs) identified in Shidalukang 1 (HR) and Junmian 1 (HS) after *Verticillium dahliae* infection, respectively. **(A)** The top 20 significant (*P* < 0.05) GO terms enriched in the 2010 and 1,275 DEGs identified in HR and HS, respectively. **(B)** The significant (*P* < 0.05) KEGG pathways of the 2010 and 1275 DEGs identified in HR and HS, respectively.

To explore the potential functionality of the DEGs identified in HR and HS, we carried out GO enrichment analyses. The 2010 DEGs in HR were significantly enriched with 114 GO terms (Supplementary Table 5). Biological processes associated with single-organism metabolic process, oxidation–reduction process and oxidoreductase activity were the most significantly enriched GO terms in HR (Figure 2A and Supplementary Table 5). In HS, the 1,275 DEGs were significantly (corrected *P* value < 0.05) enriched with 41 GO terms, including single-organism metabolic process, catalytic activity, and oxidation–reduction process (Figure 2A and Supplementary Table 6). Notably, of the top 20 GO terms enriched in HR or HS, seven overlapped between HR and HS (Figure 2A), including heme binding, oxidation–reduction process, nucleic acid binding transcription factor activity, oxidoreductase activity, transcription factor activity sequence-specific DNA binding, and tetrapyrrole binding (Figure 2A). Biological processes involved in response to endogenous stimulus and hormone were only enriched in HR (Figure 2A).

Kyoto Encyclopedia of Genes and Genomes pathway analysis results showed that the 2010 DEGs identified in HR were significantly enriched with 17 pathways, while the 1275 DEGs identified in HS were enriched with 12 pathways (Figure 2B and Supplementary Tables 7, 8). Phenylpropanoid biosynthesis, phenylalanine metabolism, alpha-linolenic acid metabolism, diterpenoid biosynthesis, monoterpenoid biosynthesis, biosynthesis of secondary metabolites, circadian rhythm-plant, sesquiterpenoid and triterpenoid biosynthesis, and nitrogen metabolism were significantly enriched in both HR and HS (Figure 2B). Pathways related to plant hormone signal transduction and flavonoid biosynthesis were significantly enriched only in HR, and the brassinosteroid (BR) biosynthesis pathway was enriched only in HS (Figure 2B).

### Differentially Expressed Genes Involved in Plant Hormone Signal Transduction

**FIGURE 3:**
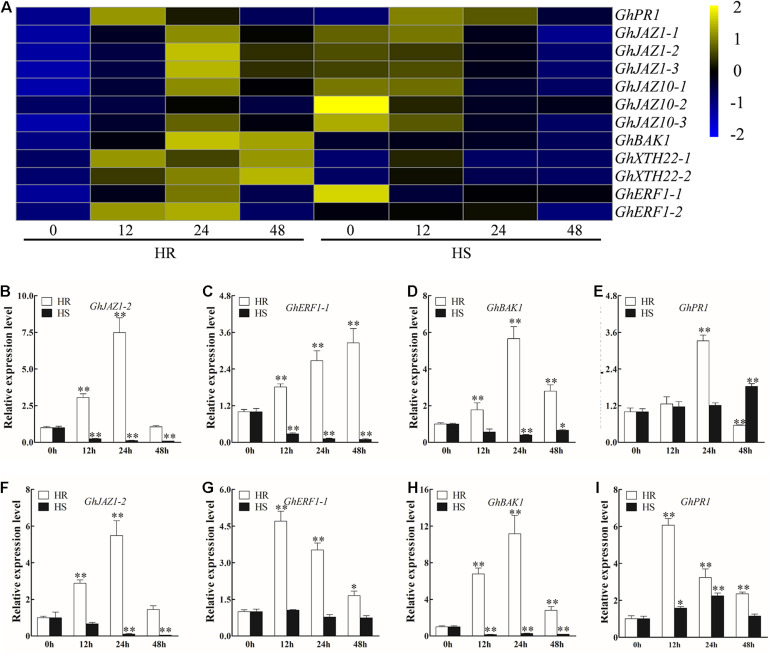
Transcriptional profile of plant hormone signal transduction-related genes after *Verticillium dahliae* infection. **(A)** Heatmap showing the expression pattern of plant hormone signal transduction-related genes in Shidalukang 1 (HR) and Junmian 1 (HS) at 0, 12, 24, and 48 h post-inoculation (hpi). The heatmap was generated using fragments per kilobase of transcript sequence per million base pairs sequenced values normalized by Z-score. **(B–E)** Relative expression levels of plant hormone signal transduction-related genes **(B)** *GhJAZ1-2*, **(C)** *GhERF1-1*, **(D)** *GhBAK1*, and **(E)** *GhPR1* in the roots of HR and HS at 0, 12, 24, and 48 hpi. **(F–I)** Relative expression levels of plant hormone signal transduction–related genes **(F)** *GhJAZ1-2*, **(G)** *GhERF1-1*, **(H)** *GhBAK1*, and **(I)** *GhPR1* in the stems of HR and HS at 0, 12, 24, and 48 hpi. Values represent mean ± SE of three independent biological replications (five individual plants per replicate). For each cultivar, the expression level at each time point was compared with that at 0 hpi that was set to 1. ^∗^ and ^∗∗^ represent significant differences from roots of the same cultivar at *P* < 0.05 and *P* < 0.01, respectively, based on Student’s *t*-test.

Previous studies have reported that plant hormones, including SA, JA, ethylene (ET), and BR, positively regulate resistance to *V. dahliae* in cotton (Shaban et al., 2018). In this study, 12 DEGs involved in SA, JA, ET, and BR signaling pathways were identified (Figure 3A and Supplementary Table 9). RNA-seq and RT-qPCR results indicated that the expression level of *GhJAZ1-2*, *GhERF1-1*, and *GhBAK1* was significantly increased in 12 to 48 hpi roots and stems of HR (except *GhJAZ1-2* at 48 hpi), but decreased in HS (Figures 3A–D,F–H). In the roots of HR, *GhPR1* showed upregulation at 24 hpi but downregulation at 48 hpi; in the stems of HR, *GhPR1* showed constant but gradually reduced upregulation from 12 to 48 hpi (Figure 3E). In HS, a significant upregulation of *GhPR1* was observed in the 48 hpi roots and 12–24 hpi stems (Figure 3I).

### Lignin Biosynthesis-Related Genes

**FIGURE 4:**
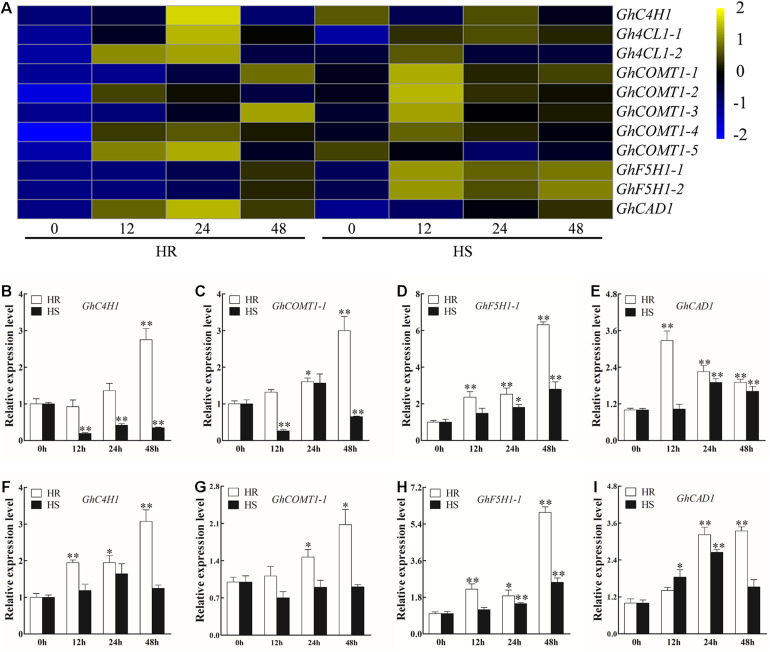
Transcriptional profile of genes involved in lignin biosynthesis after *Verticillium dahliae* infection. **(A)** Heatmap showing the expression pattern of lignin biosynthesis-related genes in HR and Junmian 1 (HS) at 0, 12, 24, and 48 h post-inoculation (hpi). The heatmap was generated using fragments per kilobase of transcript sequence per million base pairs sequenced values normalized by Z-score. **(B–E)** Real-time quantitative PCR (RT-qPCR) analysis of lignin biosynthesis-related genes **(B)** *GhC4H1*, **(C)** *GhCOMT1-1*, **(D)** *GhF5H1-1*, and **(E)** *GhCAD1* in the roots of HR and HS at 0, 12, 24, and 48 hpi. **(F–I)** RT-qPCR analysis of lignin biosynthesis-related genes **(F)** *GhC4H1*, **(G)** *GhCOMT1-1*, **(H)** *GhF5H1-1*, and **(I)** *GhCAD1* in the stems of HR and HS at 0, 12, 24, and 48 hpi. For each cultivar, the expression level at each time point was compared with that at 0 hpi that was set to 1. Values represent mean ± SE of three independent biological replications (five individual plants per replicate). ^∗^ and ^∗∗^ represent significant differences from roots of the same cultivar at *P* < 0.05 and *P* < 0.01, respectively, based on Student’s *t*-test.

Lignin has been reported to play a critical role in plant immune response to pathogens, including to *V. dahliae* (Vanholme et al., 2010; Hu et al., 2019). Transcriptome data showed that most DEGs involved in lignin biosynthesis, including two *Gh4CL*, four *GhCOMT*, two *GhF5H*, and one *GhCAD*, were increased in both HR and HS following *V. dahliae* infection, although *GhC4H1* and *GhCOMT1-5* were repressed and activated in HS and HR, respectively (Figure 4A and Supplementary Table 9). Four (*GhC4H1*, *GhCOMT1-1*, *GhF5H1-1*, and *GhCAD1*) of those 11 genes were selected for RT-qPCR verification. In agreement with the RNA-seq results, the expression level of all four genes was increased in at least one time point in the roots and stems of HR after *V. dahliae* infection. In HS, the RT-qPCR results of *GhF5H1-1*, *GhCAD1*, and *GhC4H1* were in line with the RNA-seq results (Figures 4B–I), although an inconsistent result was observed for *GhCOMT1-1* (Figures 4F,G).

### Weighted Gene Co-expression Network Analysis

**FIGURE 5:**
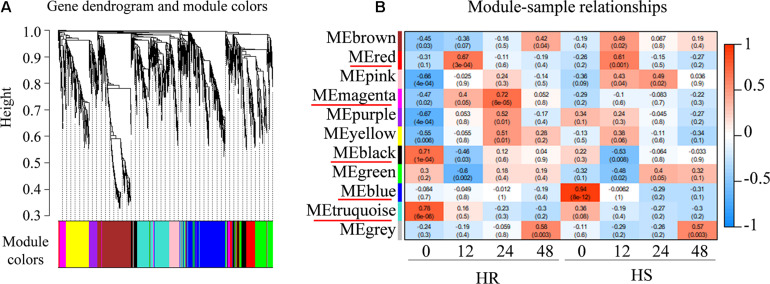
Weighted gene co-expression network analysis (WGCNA) of differentially expressed genes (DEGs) from Shidalukang 1 (HR) and Junmian 1 (HS) at 0, 12, 24, and 48 h post-inoculation. **(A)** Hierarchical cluster tree of WGCNA analysis. In total, 2,868 DEGs were grouped into 11 co-expression modules labeled with different colors based on the calculation of eigengenes. Each leaf in the tree and each major tree branch represent one gene and a distinct module, respectively. **(B)** Module–sample correlation. The color at the row–column intersection shows the level of correlation between each module and sample. The modules showing a high degree of correlation (*P* < 0.001) with samples are indicated by a red underline.

To determine the potential genes regulating cotton resistance to *V. dahliae*, 2868 non-redundant DEGs from HR and HS were subjected to WGCNA. A total of 10 gene network modules were obtained according to pairwise correlation analysis of gene expression across all samples (Figure 5A and Supplementary Table 10). As shown in Figure 4B, five module–trait relationships (indicated with red underlines) were highly significant (*P* value < 0.001) (Figure 5B). Genes in the blue module had a low expression level in HR0 while genes in the black and turquoise modules were expressed at lower levels in HS0. The transcript levels of the magenta module genes were quickly increased at 12 and 24 hpi in HR, but not significantly different after *V. dahliae* infection in HS. The transcript levels of the red module genes were rapidly increased at 12 hpi in both HR and HS (Figure 5B). In these five modules, a total of 22 hub genes related to *V. dahliae* resistance in cotton were identified using the criteria of KME > 0.9 and PPI > 5 (Table 1).

**TABLE 1:** List of the candidate hub genes in the red, magenta, black, blue, and turquoise modules.

Gene name	Description	KME value
**Blue module**		
Gh_D03G0992	Glutamate dehydrogenase 2	0.95
Gh_A08G1891	Glutamate dehydrogenase 3	0.93
**Magenta module**		
Gh_A10G1567	1-Cys peroxiredoxin	0.92
Gh_A05G2312	Stearoyl-[acyl-carrier-protein] 9-desaturase 6	0.90
**Turquoise module**		
Gh_A02G0819	Tubulin alpha-1 chain	0.95
**Red module**		
Gh_D10G0641	Photosynthetic NDH subunit of subcomplex B3	0.93
Gh_A12G0547	Ferredoxin–NADP reductase, leaf isozyme	0.91
**Black module**		
Gh_D02G1996	Chlorophyll *a*–*b* binding protein CP24 10A	0.98
Gh_A12G1617	Chlorophyll *a*–*b* binding protein P4	0.97
Gh_D12G1757	Chlorophyll *a*–*b* binding protein 4	0.97
Gh_A07G2182	Chlorophyll *a*–*b* binding protein 151	0.96
Gh_A08G0609	Photosystem I reaction center subunit II	0.96
Gh_D06G2351	Chlorophyll *a*–*b* binding protein 21	0.96
Gh_A05G2108	Chlorophyll *a*–*b* binding protein CP26	0.95
Gh_A11G2259	Chlorophyll *a*–*b* binding protein 8	0.95
Gh_D01G0531	Chlorophyll *a*–*b* binding protein 13	0.95
Gh_A05G0654	Photosystem I subunit O	0.94
Gh_A13G0222	Chlorophyll *a*–*b* binding protein 6A	0.94
Gh_D03G0876	Geranylgeranyl diphosphate reductase	0.91
Gh_D06G2350	Chlorophyll *a*–*b* binding protein 3C	0.91
Gh_A07G0818	Photosystem I reaction center subunit XI	0.91
Gh_A05G1564	Plastocyanin A	0.90

### The Expression Profiles of *GhGDH2* and *GhGDH3*

**FIGURE 6:**
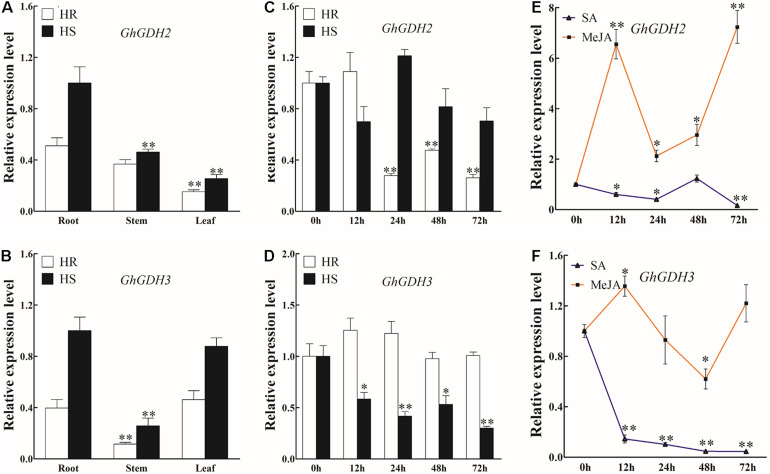
Expression profiles of *GhGDH2* in different cotton tissues and under *Verticillium dahliae*, salicylic acid (SA), or methyl jasmonate (MeJA) treatment. **(A,B)** Expression profiling of **(A)** *GhGDH2* and **(B)** *GhGDH3* in roots, stems, and leaves in Shidalukang 1 (HR) and Junmian 1 (HS). Root, stem, and leaf samples from two-leaf stage cotton seedlings were collected. **(C,D)** Expression patterns of **(C)** *GhGDH2* and **(D)** *GhGDH3* in the roots of HS and HR at 0, 12, 24, 48, and 72 h post-inoculation (hpi). **(E,F)** Expression changes of **(E)** *GhGDH2* and **(F)** *GhGDH3* in the roots of HR after treatment with SA or MeJA. Samples collected at 0, 12, 24, 48, and 72 hpi after SA or MeJA treatment were used in reverse transcription quantitative PCR. *GhUBQ7* was used as the control. Values represent mean ± SE of three independent biological replications (five individual plants per replicate). ^∗^ and ^∗∗^ represent significant differences from 0 hpi at *P* < 0.05 and *P* < 0.01, respectively, based on Student’s *t*-test.

In our previous studies with a focus on the basal transcriptome difference between HR and HS, *GhGDH3* (*Gh_A08G1891*) was characterized as a hub gene potentially associated with the defense response against *V. dahliae* infection in cotton (Xiong et al., 2021). Here, based on the genes induced or repressed by *V. dahliae* infection in HR and HS, *GhGDH3* together with one of its homologs, *GhGDH2* (*Gh_D03G0992*), were identified as hub genes of the gene networks related to *V. dahliae* response, suggesting that *GhGDH2* and *GhGDH3* may play an essential role in cotton defense against *V. dahliae* infection. To test this hypothesis, we analyzed their transcript levels in roots, stems, and leaves of uninfected HR and HS, and their response to treatment of SA or MeJA as well as to *V. dahliae* inoculation. The expression level of both *GhGDH2* and *GhGDH3* was significantly higher in HS than in HR (Figures 6A,B). In the three tissues analyzed, the transcript level of *GhGDH2* in roots was higher than that in stems and leaves in both HR and HS, while the transcript level of *GhGDH3* in the roots and leaves was significantly higher than that in stems (Figures 6A,B).

Upon *V. dahliae* infection, *GhGDH2* was significantly downregulated by 52.4 to 73.9% from 24 to 72 hpi in HR and no significant change in HS (Figure 6C), and *GhGDH3* showed an expression pattern opposite to *GhGDH2* (Figure 6D). Both *GhGDH2* and *GhGDH3* exhibited a downregulation trend after SA treatment in HR, particularly *GhGDH3*, which decreased 6.9- and 22.3-fold at 12 and 72 hpi, respectively (Figures 6E,F). *GhGDH2* and *GhGDH3* responded similarly to treatment of MeJA from 12 to 72 hpi, i.e., an initial upregulation followed by a significant downregulation and then another wave of upregulation, but with different intensity (Figures 6E,F). As a result, the expression level of *GhGDH2* at 12 to 72 hpi was significantly higher than that at 0 hpi, with an increase of 1.12- to 6.23-fold (Figure 6E), while the expression level of *GhGDH3* was significantly upregulated and downregulated at 12 and 48 hpi, respectively (Figure 6F). These results indicated that *GhGDH2* and *GhGDH3* may play a potential role in cotton defense against *V. dahliae*, which could be achieved through SA- and JA-mediated signal transduction pathways.

### Silencing of *GhGDH2* Enhanced Resistance of Cotton to *Verticillium dahliae*

**FIGURE 7:**
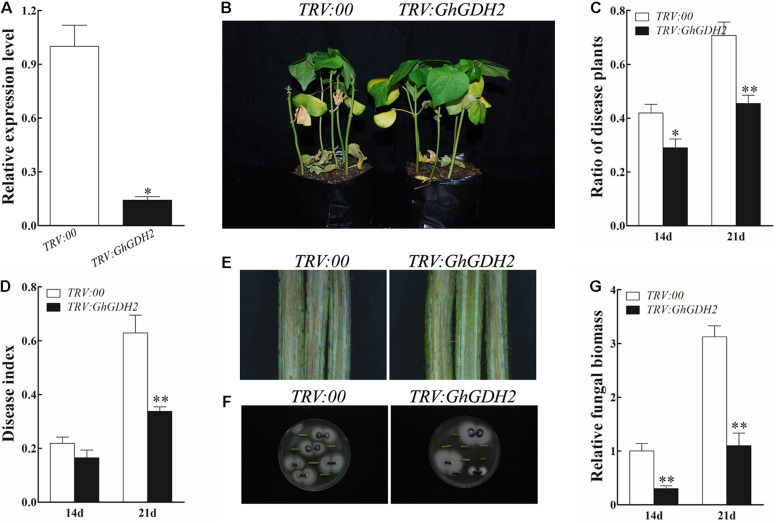
Knockdown of *GhGDH2* in *Verticillium dahliae*-susceptible cultivar Junmian 1 enhanced disease resistance. **(A)** *GhGDH2* expression in the *TRV:00* and *TRV:GhGDH2* plants. RNA was extracted from roots at 2 weeks after infiltration with the VIGS vector. **(B)** Disease phenotypes of the *TRV:00* and *TRV:GhGDH2* plants at 21 dpi. **(C,D)** Ratio of diseased plants and disease index of *TRV:00* and *TRV:GhGDH2* plants at 14 and 21 dpi. **(E,F)** Fungal recovery assay **(E)** and longitudinal sections of cotton stems **(F)** from *TRV:00* and *TRV:GhGDH2* plants at 14 dpi. **(G)** Comparison of fungal biomass. The roots from the *TRV:00* and *TRV: GhGDH2* plants at 14 and 21 dpi was used to extracted total DNA, which was used as a template for relative fungal biomass analysis by quantitative PCR analysis. Values represent mean ± SE of three independent biological replications (five individual plants per replicate). ^∗^ and ^∗∗^ represent significant differences from *TRV:00* at *P* < 0.05 and *P* < 0.01, respectively, based on Student’s *t*-test.

Given that *GhGDH2* was significantly downregulated from 24 to 72 hpi and significantly induced by MeJA in HR, we examined whether it is possible to enhance *V. dahliae* resistance of susceptible cotton by knockdown of the expression level of *GhGDH2*. To verify this conjecture, we used VIGS to knock down the expression level of *GhGDH2* in HS. The expression level of *GhGDH2* was measured using roots from *TRV:00* and *TRV:GhGDH2* plants when *TRV:GhCHLI* plants (as positive control of the VIGS experiment) exhibited the expected yellowing leaf phenotype about 3 weeks after infiltration (Supplementary Figure 2). Compared with *TRV:00* plants, *TRV:GhGDH2* plants had an approximately 82% reduction of *GhGHD2* expression (Figure 7A), and displayed less etiolated and wilted leaves at 21 dpi (Figure 7B). *TRV:GhGDH2* plants had a significantly lower ratio of diseased plants (45.5%) and disease index (33.8%) than the *TRV:00* plants (70.8 and 62.9%, respectively) at 21 dpi (Figures 7C,D). *GhGDH2*-silenced plants showed slight vascular browning in the stem at 14 dpi, whereas more severe diseased phenotype was evident in *TRV:00* plants (Figure 7E). The fungal recovery assay showed that the number of fungal colonies in stem segments from *TRV:00* plants was 20% more than that from *TRV:GhGDH2* plants (Figure 7F). *TRV:GhGDH2* plants accumulated less fungal biomass (0.3 and 1.1 at 14 and 21 dpi, respectively) than *TRV:00* plants (1.0 and 3.1 at 14 and 21 dpi, respectively) (Figure 7G). These results indicated that *GhGDH2* functions as a negative regulator of *V. dahliae* resistance.

### Downregulation of *GhGDH2* Positively Regulates JA Signaling Pathway, but Negatively Regulates SA Signaling Pathway

**FIGURE 8:**
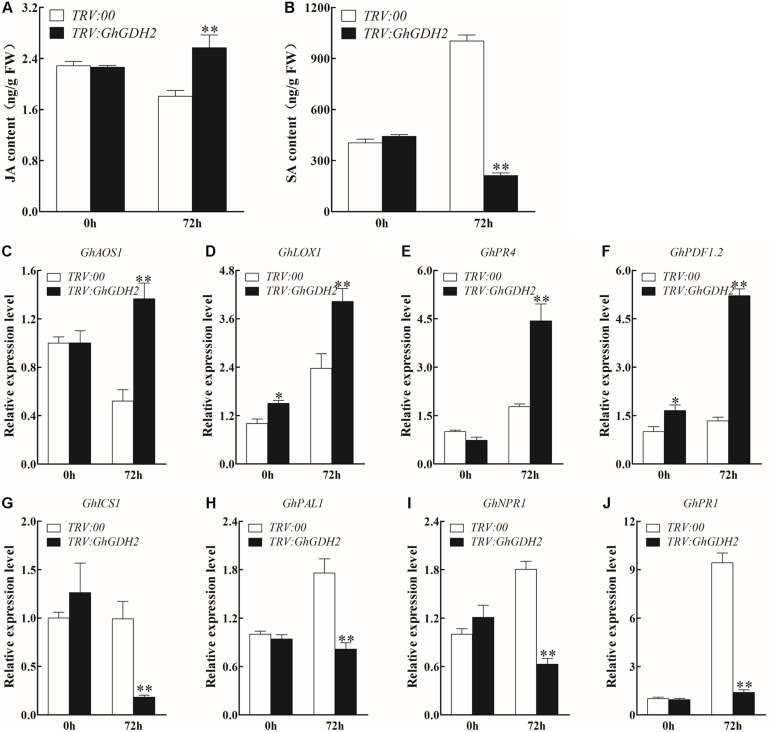
The change of jasmonic acid (JA) and salicylic (SA) signaling pathway in *GhGDH2*-silenced cotton plants. **(A,B)** Comparison of the **(A)** JA and **(B)** SA contents in *TRV:00* and *TRV:GhGDH2* plants at 72 h post-inoculation (hpi). **(C–F)** The relative expression levels of JA biosynthesis and response genes in *TRV:00* and *TRV:GhGDH2* plants at 72 hpi. **(G–J)** The relative expression level of SA biosynthesis and response genes in *TRV: 00* and *TRV:GhGDH2* plants at 72 hpi. Values represent mean ± SE of three independent biological replications (five individual plants per replicate). ^∗^ and ^∗∗^ represent significant differences from 0 h at *P* < 0.05 and *P* < 0.01, respectively, based on Student’s *t*-test.

To explore whether the improvement of *V. dahliae* resistance in *GhGDH2*-silenced plants is mediated by JA and SA signaling pathways, we measured JA and SA concentrations in the roots of *TRV:00* and *TRV:GhGDH2* plants with or without *V. dahliae* inoculation. No significant difference in the content of JA and SA was detected between *TRV:GhGDH2* and *TRV:00* plants under the mock treatment (Figures 8A,B). At 72 hpi, the content of JA and SA in *TRV:GhGDH2* plants increased and decreased 42.1 and 78.9%, respectively, compared with that in *TRV:00* plants (Figures 8A,B). Consistent with this result, JA biosynthesis genes (*GhAOS1* and *GhLOX1*) and JA response genes (*GhPR4* and *GhPDF1.2*) were significantly induced in *TRV:GhGDH2* plants at 72 hpi (Figures 8C–F), while the expression levels of SA biosynthesis (*GhPAL1* and *GhICS1*) and response genes (*GhNPR1* and *GhPR1*) were significantly downregulated in *TRV:GhGDH2* plants at 72 hpi (Figures 8G–J). These results were consistent with those observed in HR (Figures 6C,E) and support the notion that *GhGDH2* plays a negative role in *V. dahliae* resistance by activating JA signaling pathway and repressing SA signaling pathway.

## Discussion

### Hub Genes for *Verticillium dahliae* Resistance in Cotton

Mining *V. dahliae*-resistant genes is of great importance to breed disease-resistant cotton cultivars. We have used comparative transcriptome analysis to identify genes differentially expressed between Upland cotton cultivars resistant (HR) or susceptible (HS) to *V. dahliae* under uninfected or infected conditions. DEGs were subjected to investigate gene networks and hub genes associated with disease resistance. While our previous study was focused on the basal difference between HR and HS (Xiong et al., 2021), this study explored the transcriptomic responses of HR and HS upon *V. dahliae* infection. WGCNA is an effective method that have been previously used to identify hub genes involved in various biological processes and disease resistance in many plant species, such as soybeans (Du et al., 2017), rice (Tan et al., 2017), and cotton (Sun et al., 2019; Xiong et al., 2021). In this study, 22 hub genes related to *V. dahliae* resistance were identified based on WGCNA, including two genes encoding GDH (Figure 5B and Table 1).

Glutamate dehydrogenase is a key enzyme catalyzing the reversible reduction of glutamate into 2-oxoglutarate, which plays vital roles in plant amino acid metabolism (Fontaine et al., 2012; Seifi et al., 2013; Tsai et al., 2016). Studies have reported induction of *GDHs* by pathogen invasion in sunflower (Dulermo et al., 2009), tobacco (Pageau et al., 2006), and common bean (Tavernier et al., 2007). On the basis of identification of 22 hub genes (Figure 5B and Table 1), we analyzed the expression patterns of *GhGDH2* and *GhGDH3* after *V. dahliae* infection, or treatment of SA or MeJA (Figure 6), and found that *GhGDH2* negatively regulates *V. dahliae* resistance in cotton, which was confirmed by the finding that knockdown of the expression level of *GhGDH2* in cultivar susceptible to *V. dahliae* was able to enhance disease resistance (Figure 7). Therefore, *GhGDH2* is a candidate gene for breeding cotton cultivars’ resistance to *V. dahliae* by downregulating its expression in susceptible elite cultivars through RNAi or gene editing. Gene editing would be the ideal strategy as the dysfunctional *GhGDH2* created by the technology can be transferred to any other cultivars by crossing. *GDH* genes have been reported to be engaged in plant defense responses to pathogen infections (Pageau et al., 2006; Tavernier et al., 2007; Dulermo et al., 2009). Here, we found that *GhGDH3* of cotton is also potentially involved in the response to *V. dahliae* infection but may have a function different from *GhGDH2* as it showed downregulation in cultivar susceptible to *V. dahliae* infection (Figure 6D) and was repressed by MeJA at 48 hpi (Figure 6F). It is of our interest to further explore the potential role of *GhGDH3* in regulating *V. dahliae* resistance.

In cotton, several studies have associated activating JA signaling pathways with *V. dahliae* resistance (Li et al., 2014; Sun et al., 2014; Xiong et al., 2019). Our results support those previous results that the expression of genes related to JA biosynthesis and response were significantly increased in *TRV:GhGDH2* plants than in *TRV:00* plants, as well as JA content at 72 hpi, and showed an enhanced *V. dahliae* resistance (Figures 8A,C–F). JA and SA often showed an antagonistic interaction in plant defense (Durrant and Dong, 2004; Thaler et al., 2012). In general, accumulation of JA content results in decreased level of SA and inhibition of SA signaling pathways after pathogen infection (Shim et al., 2013). We have previously observed this phenomenon in cotton for *GhWRKY70A05* (Xiong et al., 2019); here, we demonstrated another such example in which downregulation of *GhGDH2* promoted JA signaling pathways, and inhibited SA biosynthesis (Figure 8B) and signaling pathways (Figures 8G–J) upon *V. dahliae* infection, consequently improving *V. dahliae* resistance in cotton.

In addition to *GDH* genes, several other hub genes are of interest. Stearoyl-[acyl-carrier-protein]-desaturase (SAD) is involved in biosynthesis of fatty acids and produces monounsaturated fatty acids in plant cells (Kachroo et al., 2007). There are seven *SAD* genes in the *Arabidopsis* genome, some of which have been recognized to mediate plant defense response by regulating SA and JA signaling pathways (Kachroo et al., 2003, 2007). In our study, *GhSAD6* (*Gh_A05G2312*), showing 73% amino acid sequence identity with *AtSAD6*, was one of the hub genes in the magenta module (Table 1). The expression level of *GhSAD6* was significantly induced following *V. dahliae* infection (Figure 5B). Previously, a large number of photosynthesis-related gene have been reported to be downregulated by pathogens (de Torres Zabala et al., 2015). Our KEGG pathway analysis found that genes involved in photosynthesis were significantly enriched in HR, and their expression was significantly downregulated after *V. dahliae* infection in HR (Figure 2B and Supplementary Table 7). In the black module, several genes related to photosynthesis were identified as hub genes, including 10 chlorophyll *a*–*b* binding proteins, two photosystems I reaction center subunit and one photosystem I subunit O, and two photosystems I reaction center subunit (Table 1). These results indicate that the *V. dahliae* resistance observed in HR may be partly due to its better ability (compared with HS) to slow down growth and to divert more energy to defense, as downregulation of the metabolic pathways related to plant growth and development contributes to plant defense against pathogens (Attaran et al., 2014; Huot et al., 2014; Dhar et al., 2020).

### Cotton Resistance to *Verticillium dahliae* Is Co-regulated by Various Plant Hormones

Jasmonic acid, SA, and ET are three important signaling molecules crucial for modulating plant defense response to pathogens (Koornneef and Pieterse, 2008), including *V. dahliae* (Dhar et al., 2020). Recent studies showed that JA signaling pathway positively regulates *V. dahliae* resistance in cotton (Gao et al., 2013a; Xiong et al., 2019). Two JAZ genes (*GbJAZ1* and *GbJAZ10*) which act as repressors of JA signaling pathways are downregulated in *G. barbadense* cv Hai7124 after *V. dahliae* infection (Xu et al., 2011b). Different from this result, upregulated and downregulated expressions of three *GhJAZ1* and three *GhJAZ10* were found in HR and HS after *V. dahliae* infection, respectively (Figure 3A and Supplementary Table 9). One possible reason is that JA signaling pathway may have different regulatory functions in *G. hirsutum* and *G. barbadense* regarding their role in resistance to *V. dahliae* (Duan et al., 2016). RT-qPCR analyses revealed that the relative expression of a SA response gene *GhPR1* was significantly induced in both resistant (HR) and susceptible (HS) varieties upon *V. dahliae* infection (Figures 3A,E,I), indicating that SA signaling pathway was activated by *V. dahliae* infection. A similar result has been reported in a previous study (Zhang et al., 2013). The upregulation of three *GhJAZ1* and three *GhJAZ10* in HR might be a result of the antagonistic interaction between the SA and JA signaling pathways (Durrant and Dong, 2004). Meanwhile, *GhJAZ2* was also upregulated in *G. hirsutum* and negatively regulated cotton resistance to *V. dahliae* (He et al., 2018). These studies imply that *GhJAZ1* and *GhJAZ10* might be pivotal genes mediating cotton defense response against *V. dahliae* infection.

Ethylene is the only plant gas hormone that has been reported to be involved in various biological processes in plants. It often interacts synergistically with JA to advance resistance against necrotrophic pathogens (Zhu et al., 2011). *ERF* genes are important downstream response factors of the ET signaling pathway and participated in plant responses to pathogens (Amorim et al., 2016). Several reports have demonstrated upregulation of the *ERF* expression in cotton after *V*. *dahliae* infection (Wang et al., 2011; Xu et al., 2011a; Guo et al., 2016). Consistent with those results, upregulation and downregulation of three *ERF* genes were found in HR and HS after *V. dahliae* infection, respectively (Figure 3A and Supplementary Table 9). Moreover, knockdown of *GhERF1-like* and *GhERF6* significantly decreases *V. dahliae* resistance in cotton by inhibiting the ET signaling pathway (Yang et al., 2015; Guo et al., 2016). Thus, the activation of ERF in the ET signaling pathway is beneficial to improve cotton resistance to *V. dahliae*.

Besides JA, SA, and ET, BR also is an important plant hormone, which has been reported to participate in the process of cotton defense against *V. dahliae* infection (Gao et al., 2013a; Shaban et al., 2018). BAK1, a leucine-rich-repeat receptor-like protein kinase, has been recognized to modulate BR signaling pathway (Li et al., 2002). In *Arabidopsis*, *bak1-4* mutant plants are more susceptible to *V. dahliae* (Fradin et al., 2011). Silencing the expression of *BAK1* through VIGS significantly decreases cotton resistance to *V. dahliae* (Gao et al., 2013b). Our results showed that several genes involved in the BR signaling pathway, for example, one *BAK1* gene, were increased in HR but decreased in HS after *V. dahliae* infection (Figures 3A,D,H and Supplementary Table 9), suggesting that BR might have positive regulatory effects upon *V. dahliae* infection.

### Lignin Biosynthesis in Cotton Is Activated by *Verticillium dahliae* Infection

Kyoto Encyclopedia of Genes and Genomes pathway analysis indicated that phenylpropanoid biosynthesis is potentially implicated in defense against *V. dahliae* infection (Figure 2B). Lignin is biosynthesized by a main branch of the phenylpropanoid biosynthesis pathway and has a significant role in plant defense (Boerjan et al., 2003). Phenylalanine-ammonia lyase (PAL) and peroxidase are two important enzymes in the process of lignin biosynthesis, and their enzymatic activities were significantly increased in the Verticillium wilt-resistant tomato variety after *V. dahliae* infection (Yang et al., 2018; Hu et al., 2019). Studies have also reported the induction of lignin biosynthesis-related genes by pathogen invasion in *Arabidopsis* (Wang et al., 2018) and eggplant (Zhou et al., 2016). In the *V. dahliae*-resistant *G. barbadense* cv Hai7124, *V. dahliae* infection results in the upregulation of a large number of lignin biosynthesis-related genes including *PAL*, *4CL*, *F5H*, *CCoAOMT*, and *CAD* (Xu et al., 2011b). In another *V. dahliae*-resistant *G. barbadense* cv Xinhai 15, the expression level of *COMT* is induced by *V. dahliae* (Zhang et al., 2013). We also found that 13 upregulated DEGs in HR were involved in lignin biosynthesis, such as *C4H*, *4CL*, *COMT*, *F5H*, and *CAD* (Figure 4A and Supplementary Table 9). This finding is consistent with those of Zhang et al. (2013), indicating a role of genes related to lignin biosynthesis in *V. dahliae* resistance. Furthermore, numerous disease resistance genes positively regulate cotton resistance to *V. dahliae* by promoting lignin biosynthesis (Shaban et al., 2018). However, of the key genes involved in lignin biosynthesis, only *Gh4CL30* and *GhHCT1* have been functionally characterized in the context of *V. dahliae* infection (Guo et al., 2016; Xiong et al., 2021), the functions of other lignin biosynthesis-related genes in cotton resistance to *V. dahliae* are yet to be studied.

## Conclusion

This study conducted a comparative transcriptomic analysis using *V. dahliae*-resistant (HR) and *V. dahliae*-susceptible (HS) cotton cultivar uninfected or infected with *V. dahliae*, and identified DEGs after *V. dahliae* infection in HR and HS. Our results support the notion of the importance of lignin and plant hormones in cotton defense against *V. dahliae* infection. Importantly, we demonstrated that *GhGDH2* negatively modulate cotton resistance to *V. dahliae* and that it was possible to increase *V. dahliae* resistance of susceptible cotton cultivar by downregulating the expression level of *GhGDH2*. Our results also showed WGCNA being a particularly useful approach in identifying gene modules and candidate hub genes contributing to *V. dahliae* resistance.

## Data Availability Statement

The datasets presented in this study can be found in online repositories. The names of the repository/repositories and accession number(s) can be found in the article/Supplementary Material.

## Author Contributions

JS and FX conceived and designed the experiments. X-PX. performed the experiments and wrote the manuscript. S-CS performed the experiments and analysis of RNA-seq data. Q-HZ revised the manuscript. FL, X-YZ, and Y-JL performed the experiments. All authors contributed to the article and approved the submitted version.

## Conflict of Interest

The authors declare that the research was conducted in the absence of any commercial or financial relationships that could be construed as a potential conflict of interest.

## Footnotes

1 https://magic.novogene.com

2 https://biodb.swu.edu.cn/qprimerdb/

## Supplementary Material

Supplementary Figure 1 Venn diagram showing shared or unique differentially expressed genes in Shidalukang 1 (HR) and Junmian 1 (HS) after *Verticillium dahliae* infection.

Supplementary Figure 2 Phenotypes of *GhCHLI* interference effect in *Verticillium dahliae*-susceptible cultivar Junmian 1 at 3 weeks after infiltration with *Agrobacterium.*

supplementary Table 1 All primers used in this study.

supplementary Table 2 Differentially expressed genes in Shidalukang 1 at 12, 24, and 48 h after *Verticillium dahliae* infection.

Supplementary Table 3 Differentially expressed genes in Junmian 1 at 12, 24, and 48 h after *Verticillium dahliae* infection.

Supplementary Table 4 Number of all differentially expressed genes induced by *Verticillium dahliae* in Shidalukang 1 and Junmian 1 at different time points.

Supplementary Table 5 Gene ontology analysis of induced and repressed genes in Shidalukang 1 after infection with *Verticillium dahliae*.

Supplementary Table 6 Gene ontology analysis of induced and repressed genes in Junmian 1 after infection with *Verticillium dahliae*.

Supplementary Table 7 Kyoto Encyclopedia of Genes and Genomes pathways analysis of induced and repressed genes in Shidalukang 1 after infection with *Verticillium dahliae*.

Supplementary Table 8 Kyoto Encyclopedia of Genes and Genomes pathways analysis of induced and repressed genes in Junmian 1 after *Verticillium dahliae* infection.

Supplementary Table 9 The expression patterns of genes involved in the lignin biosynthesis and plant hormone signal transduction in Shidalukang 1 and Junmian 1 after *Verticillium dahliae* infection.

Supplementary Table 10 List of all genes for each module.
