# Supplementary material for: Transcriptome Analysis and RNA Interference Reveal GhGDH2 Regulating Cotton Resistance to Verticillium Wilt by JA and SA Signaling Pathways
Source: Front Plant Sci. 2021 Jun 11;12:654676. doi: 10.3389/fpls.2021.654676 (PMC8226099; doi:10.3389/fpls.2021.654676)

Fig. S1. Venn diagram showing shared or unique differentially expressed genes in Shidalukang 1(HR) and Junmian 1 (HS) following *Verticillium dahliae* infection.

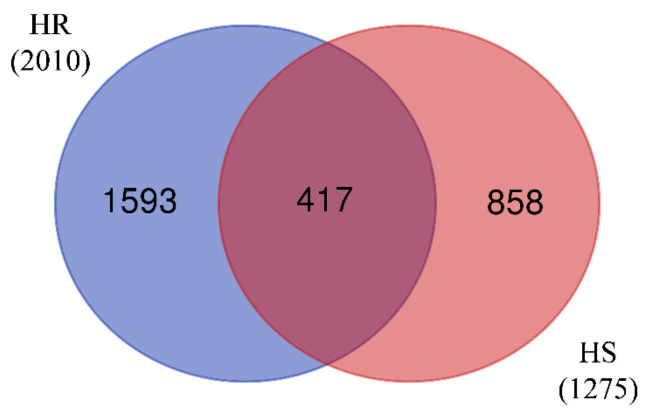

Fig. S2. Phenotypes of *GhCHLI* interference effect in *Verticillium dahliae*-susceptible cultivar Junmian 1 at three weeks after infiltration with *Agrobacterium.*

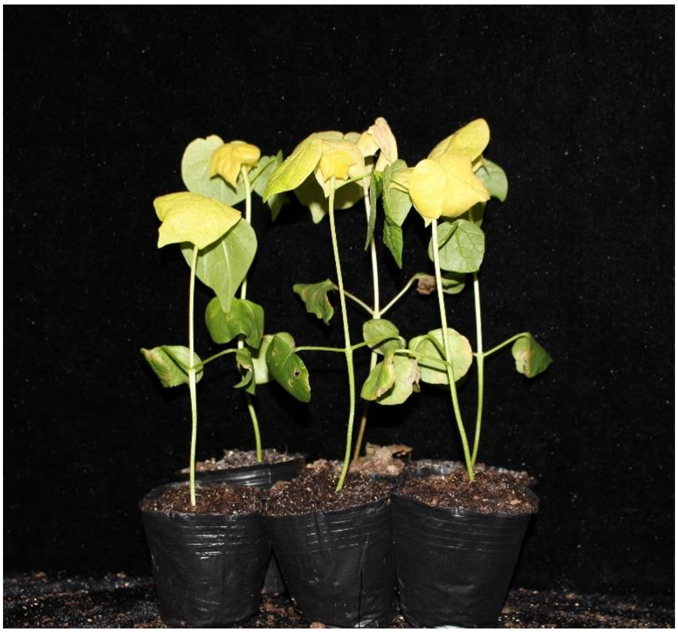

Supplement: Supplementary Figure 1 — Venn diagram showing shared or unique differentially expressed genes in Shidalukang 1 (HR) and Junmian 1 (HS) after Verticillium dahliae infection. [file Data_Sheet_1.PDF]
